# Sodium Butyrate Induces Endoplasmic Reticulum Stress and Autophagy in Colorectal Cells: Implications for Apoptosis

**DOI:** 10.1371/journal.pone.0147218

**Published:** 2016-01-19

**Authors:** Jintao Zhang, Man Yi, Longying Zha, Siqiang Chen, Zhijia Li, Cheng Li, Mingxing Gong, Hong Deng, Xinwei Chu, Jiehua Chen, Zheqing Zhang, Limei Mao, Suxia Sun

**Affiliations:** 1 Department of Nutrition and Food Hygiene, Guangdong Provincial Key Laboratory of Tropical Disease Research, School of Public Health, Southern Medical University, No.1023 South Sha-Tai Rd, Guangzhou, Guangdong, P.R.China, 510515; 2 Department of Certification Supervision, Guangdong Entry-Exit Inspection and Quarantine Bureau, Guojian Building, No.66, Huacheng Avenue, Zhujiang Xincheng, Guangzhou, Guangdong Province, P.R. China 510623; Taipei Medicine University, TAIWAN

## Abstract

**Purpose:**

Butyrate, a short-chain fatty acid derived from dietary fiber, inhibits proliferation and induces cell death in colorectal cancer cells. However, clinical trials have shown mixed results regarding the anti-tumor activities of butyrate. We have previously shown that sodium butyrate increases endoplasmic reticulum stress by altering intracellular calcium levels, a well-known autophagy trigger. Here, we investigated whether sodium butyrate-induced endoplasmic reticulum stress mediated autophagy, and whether there was crosstalk between autophagy and the sodium butyrate-induced apoptotic response in human colorectal cancer cells.

**Methods:**

Human colorectal cancer cell lines (HCT-116 and HT-29) were treated with sodium butyrate at concentrations ranging from 0.5–5mM. Cell proliferation was assessed using MTT tetrazolium salt formation. Autophagy induction was confirmed through a combination of Western blotting for associated proteins, acridine orange staining for acidic vesicles, detection of autolysosomes (MDC staining), and electron microscopy. Apoptosis was quantified by flow cytometry using standard annexinV/propidium iodide staining and by assessing PARP-1 cleavage by Western blot.

**Results:**

Sodium butyrate suppressed colorectal cancer cell proliferation, induced autophagy, and resulted in apoptotic cell death. The induction of autophagy was supported by the accumulation of acidic vesicular organelles and autolysosomes, and the expression of autophagy-associated proteins, including microtubule-associated protein II light chain 3 (LC3-II), beclin-1, and autophagocytosis-associated protein (Atg)3. The autophagy inhibitors 3-methyladenine (3-MA) and chloroquine inhibited sodium butyrate induced autophagy. Furthermore, sodium butyrate treatment markedly enhanced the expression of endoplasmic reticulum stress-associated proteins, including BIP, CHOP, PDI, and IRE-1a. When endoplasmic reticulum stress was inhibited by pharmacological (cycloheximide and mithramycin) and genetic (siRNA targeting BIP and CHOP) methods, the induction of BIP, PDI, IRE1a, and LC3-II was blocked, but PARP cleavage was markedly enhanced.

**Discussion:**

Taken together, these results suggested that sodium butyrate-induced autophagy was mediated by endoplasmic reticulum stress, and that preventing autophagy by blocking the endoplasmic reticulum stress response enhanced sodium butyrate-induced apoptosis. These results provide novel insights into the anti-tumor mechanisms of butyric acid.

## Introduction

Colorectal cancer (CRC) is the third most common cancer and the fourth leading cause of cancer-related death worldwide. In 2008, there were an estimated 1,233,700 new cases and 608,700 deaths [1]. Despite the advent of targeted therapies (e.g. cetuximab and bevacizumab), and improvements in other treatment modalities, the prognosis for patients with metastatic CRC remains poor [2]. Thus, there is an urgent need to develop new chemoprophylactic agents to prevent CRC at the early stages.

The role of a high fiber diet in preventing some forms of cancer has been recognized for many years [3]. Short-chain fatty acids (SCFAs) are the major by-products of bacterial fermentation of undigested dietary fibers in the human colon [4]. SCFAs have been shown to have anti-tumor effects related to induction of tumor cell death, and are currently being investigated as adjuvant therapies for colorectal cancer [5]. The three major SCFAs—acetate (2C), propionate (3C), and butyrate (4C)—are found in the colon at a molar ratio of 60:25:15, respectively [6]. Physiological concentrations of total SCFAs in the human intestines range from 70–140mM in proximal colon to 20–70mM in the distal colon to 20–40mM in the distal ileum [7, 8]. Interestingly, the type of cell death triggered by SCFAs is different depending on the carbon length. For example, butyrate preferentially induces apoptosis or necrosis compared to propionate in gastric cancer cells [5]. Butyrate has received the most attention as an anti-tumor treatment due to its potent and pleiotropic anticancer effects. Butyrate has been shown to trigger high rates of apoptosis in HT-29 CRC cells in vitro, and to reduce the invasive properties of primary adenocarcinoma CRC cells [5]. Sodium butyrate (NaB) inhibits cell cycle progression, promotes differentiation, and induces apoptosis and autophagy in several types of cancer cells, including CRC, lymphoma, and breast cancer cells [9–12]. However, the mechanism of action remains elusive [5]. It has been suggested that butyrate is the preferred energy source for normal colonocytes and supports homeostasis [13], but butyrate accumulates in cancerous colonocytes due to the Warburg effect where it functions as an HDAC inhibitor, inhibiting cell proliferation and stimulating apoptosis [14–16]. It is feasible that butyrate may function not only as a preventative agent in the colon of people who routinely eat fiber-rich diets, but also as a therapeutic agent, especially in people who typically had eaten diets with low fiber content but subsequently changed to fiber-rich diets.

Multiple cell death pathways can activate in tumor cells. Apoptosis is the canonical cell death pathway. Apoptosis can be triggered by external stimuli engaging the “death receptors” on the cell surface (e.g. Fas) or by internal stimuli in response to cellular stress (e.g. mitochondrial or endoplasmic reticulum [ER] damage) [17]. Apoptosis is a highly regulated process that is mediated by the caspase family of proteases. The ER and mitochondria are both involved in the intracellular stress response, which leads to changes in the concentration of calcium cations (Ca^2+^) and caspase activation [17]. Autophagy is a lysosome-dependent pathway involved in the turnover of cellular macromolecules and organelles [18]. Autophagy is characterized by the formation of double membrane vesicles that carry cargo eventually destined for lysosomal degradation [19]. It is a highly conserved multi-step process that is regulated by several ‘Atg’ (Autophagy-related) genes [20]. The initial nucleation and assembly of the primary autophagosomal membrane requires a kinase complex that contains class III phosphatidylinositol 3-kinase (PI3K) and beclin 1 [21]. The isolation membrane elongates to enclose cellular contents, a process mediated by two ubiquitin-like conjugation systems. One of the ubiquitin-like systems converts microtubule-associated protein 1 light chain 3 (LC3) from the free form (LC3-I) to a lipid-conjugated membrane-bound form (LC3-II). The accumulation of LC3-II and its localization to vesicular structures are commonly used as markers of autophagy [22]. The role of autophagy in tumor cells is unclear. It has paradoxically been shown to be both a pro-survival and a pro-death pathway depending on context [19]. For example, hemizygous loss of beclin 1 has been shown to increase the incidence of tumors in both mice and humans, likely due to the accumulation of cells with genomic instability that would otherwise have been eliminated through autophagy] 19]. However, autophagy can also be activated as a survival pathway in conditions where cells are metabolically stressed (e.g. hypoxia), allowing cells to scavenge intracellular macromolecules to promote survival in a quiescent state, and to avoid necrotic cell death brought on by nutrient deprivation [19].

The ER is involved in biosynthesis, protein folding, free calcium storage, and modification of various soluble and insoluble proteins [23]. The ER is very sensitive to minor perturbations in its environment such as glucose deprivation, oxidative stress, and infection. ER disturbances can lead to the accumulation of misfolded or unfolded proteins, which triggers an unfolded protein response (UPR), more generally referred to as ER stress [24]. Numerous studies have suggested that ER stress leads to both autophagy and apoptosis [21, 25, 26]. Release of calcium from the ER activates calcium- dependent kinase kinase-β (CaMKKβ) and AMPK, which inhibit mTOR, thereby promoting autophagy [21]. We have previously shown that NaB induces calcium release from the ER, which in turn causes an extracellular calcium influx in colorectal cancer cells [27]. Therefore, we hypothesized that ER stress may be triggering NaB-induced autophagy in CRC cells. The aim of this study was to establish whether the NaB treatment was associated with ER stress and autophagy induction in two different types of CRC: colorectal carcinoma and colorectal adenocarcinoma.

## Materials and Methods

### Reagents and antibodies

BIP, CHOP small interference (siRNA), and control siRNA were purchased from GenePharma (ShangHai, China). Fetal bovine serum (FBS), Dulbecco’s modified eagle medium (DMEM), and antibiotics were purchased from Gibco-Invitrogen (Carlsbad, CA, USA). Sodium butyrate, defatted BSA (BSA), not-fat milk, monodansylcadaverin (MDC), acridine orange (AO), 3-methyladenine (3-MA), chloroquine, cycloheximide, mithramycin, 3-(4,5-dimethyl-2-thiazolyl)-2,5-diphenyl-2-H-tetrazolium bromide (MTT), and other chemicals were purchased from Sigma-Aldrich (St. Louis, MO, USA). LC3B, ATG3, Beclin 1, CHOP, IRE-1a, BIP, PARP, and GAPDH antibodies were purchased from Cell Signaling Technology (Beverly, MA, USA). All of the other chemicals and reagents were purchased from Sigma-Aldrich (St. Louis, MO, USA) unless otherwise specified.

### Cell culture

The human colorectal carcinoma HCT-116 and the colorectal adenocarcinoma HT-29 cell lines were obtained from the American Type Culture Collection (Manassas, VA, USA). The cells were grown in DMEM supplemented with 10% FBS, penicillin (100 U/mL), and streptomycin (100 μg/mL) at 37°C in 5% CO_2_.

### MTT assay

HCT-116 and HT-29 cells were plated at 1×10^4^ cells per well in a 200 μL volume in 96-well plates. Cells were then treated as indicated and the 3-(4, 5-dimethylthiazol- 2-yl)-2, 5-diphenyltetrazoliumbromide (MTT) solution was added to the culture medium at a final concentration of 0.5mM in each well. The plates were incubated for an additional 4 h at 37°C. Dimethyl sulfoxide (DMSO) was added to solubilize the MTT tetrazolium crystals. Finally, the optical density was determined at 490nm using a plate reader (BioTek Instruments, USA). The percentage of proliferation was calculated as follows:

Proliferation rate(%)=A490(sample)/A490(control)×100%

### Apoptosis assays

Phosphatidylserine externalization and PARP cleavage were used to evaluate apoptosis. An annexinV/PI (phosphoinositide) apoptosis detection kit was used to detect externalization of phosphatidylserine according to the manufacturer’s instructions (BD Pharmingen). In brief, 1×10^6^ HCT-116 cells were washed twice with PBS and stained with 5 μL of annexin V-FITC for and 10 μL of PI in binding buffer (10 mM Hepes, pH 7.4, 140 mM NaOH, and 2.5 mM CaCl_2_) for 15 min at 4°C. Flow cytometric analysis was conducted on a FACScan using the Cell Quest software package (Becton Dickinson). Apoptotic cells were defined as annexinV+/PI-.

### Acridine orange staining for acidic vesicular organelles

Cells were stained with acridine orange using previously published procedures [28]. Briefly, acridine orange was added at a final concentration of 1 mg/mL for 20 min. Images were obtained using a fluorescence microscope (Olympus, IX-73) equipped with a mercury 100-W lamp, 460-495-nm band-pass blue excitation filters, a 505-nm dichroic mirror, a 510-nm long pass-barrier filter, and a digital camera (Olympus DP80).

Acridine orange stained cells were also used to quantify the volume of the acidic components in the cell. In acridine orange-stained cells, the cytoplasm and nucleolus fluoresce bright green and dim red, however acidic compartments fluoresce bright red [28, 29]. The intensity of the red fluorescence is proportional to the degree of acidity. Therefore, the volume of the cellular acidic compartment can be quantified based on the intensity of the red fluorescence [28, 30]. Cells were stained with acridine orange for 20 min, removed from the plate with trypsin-EDTA, and collected in phenol red-free growth medium. Green (510–530 nm) and red (650 nm) fluorescence emission from 1×10^4^ cells excited with blue (488 nm) light was measured using a FACSCanto^TM^ II from (Becton Dickinson) and the CellQuest software package.

### Monodansylcadaverine (MDC) staining for autolysosomes

HCT-116 and HT-29 cells were stained with a 0.05 mM final concentration of MDC (D4008) in PBS for 30 min at 37°C. Cells were then washed with PBS two times to remove excess MDC and fixed with 4% (v/v) paraformaldehyde (P6148) for 30 min. Images were obtained using a fluorescence microscope (Olympus DP80) equipped with a 340-390-nm band-pass ultraviolet excitation filter, a 410-nm dichroic mirror, and a 420-nm long pass-barrier filter.

### Electron microscopy

HCT-116 cells were harvested by trypsinization, washed twice with PBS, and fixed with ice-cold glutaraldehyde [2.5% glutaraldehyde in PBS (pH 7.4)] for 30min. The cells were washed with PBS, fixed in osmium tetraoxide (OsO_4_), and embedded in Spurr's Epon. Representative areas were chosen for ultra-thin sectioning and viewed with a Hitachi 7500 electron microscope.

### Western blotting

Western blots were performed as previously described. Briefly, HCT-116 and HT-29 cells were lysed for 30 min with ice-cold lysis buffer (50 mM Tris-HCl, [pH 7.5], 150 mM NaCl, 0.5% cholate acid, 0.1% SDS, 2 mM EDTA, 1% Triton X-100, and 10% glycerol) containing a commercial protease inhibitor cocktail (Roche, Mannheim, Germany). Total cell lysates were centrifuged at 14,000× g for 15 min at 4°C. The supernatant was collected, and the protein concentration was determined with a Bradford assay kit (Bio-Rad Laboratories, Hercules, CA, USA). Equal amounts of protein (30 μg/lane) were loaded and separated by 10% SDS-PAGE, then transferred to PVDF membranes (Bio-Rad Laboratories), and incubated with primary antibodies at 4°C overnight. Horseradish peroxidase-conjugated goat anti-mouse IgG was used as a secondary antibody. The specific complexes were visualized using the supersignal west pico chemiluminescent substrate detection kit (Pierce Biotechnology) according to manufacturer’s instructions. The level of protein expression was quantified with the Optiquant version 3.00 (Packard instrument Co.) program and normalized to the GAPDH (protein of interest:GADPH). The semi-quantitative results of the WB technique can be influenced by loading quantity of sample, the concentration of the primary antibodies and second antibodies, and developing time. Analysis of gray was used to quantitate the results of the experiments. The fold change from GADPH control was expressed as mean ± standard deviation (SD) of three independent experiments. Thus, the shown single raw WB image may not exactly match the mean ± standard deviation (SD) of the quantified results of three independent experiments.

### Real-time quantitative PCR

After appropriate treatments to HCT-116 and HT-29 cells in 6-well plates, RNA was extracted by using TRIzol (Invitrogen, 15596–026), and 500ng of RNA was reverse-transcribed to cDNA. Beclin1, ATG3 and LC3B mRNA level was determined by using PrimeScript RT reagent Kit (Perfect Real Time) (TAKARA, RR037A) and the appropriate primers listed in S1 Table. Samples (100 ng cDNA) were amplified in triplicate by real-time polymerase chain reaction (PCR) with the conditions: 95°C for 30 sec, 40 cycles of (5 sec at 95°C, 30 sec at 60°C), 95°C for 30 sec, and 55°C for 30 sec, followed by melting curves from 55° to 95°C.

### RNA interference

To silence gene expression by RNA interference, HCT-116 and HT-29 cells were transfected with siRNAs using Lipofectamine 2000 (Invitrogen, Carlsbad, CA) according to the manufacturer’s instructions. The following siRNA sequences were used to target the human genes of interest:

BIP-1 sense: 5'-AGUGUUGGAAGAUUCUGAUTT-3',

antisense: 5'-AUCAGAAUCUUCCAACACUTT-3';

BIP siRNA-2 sense: 5'-GGAGCGCAUUGAUACUAGATT-3',

antisense, 5'-UCUAGUAUCAAUGCGCUCCTT-3';

CHOP-1 sense: 5'-CUACCAGAAAGGUAUACCUTT-3',

antisense: 5'-AGGUAUACCUUUCUGGUAGTT-3'; and

CHOP siRNA-2 sense: 5'- GACCCUUGUGCUCGUUGUCTT-3',

antisense: 5'-GACAACGAGCACAAGGGUCTT-3'.

The scrambled negative control siRNA sequences were sense: 5'-UUCUCCGAACGUGUCACGUTT-3' and antisense: 5'-ACGUGACACGUUCGGAGAATT-3'.

### Statistical analysis

All experiments were performed in triplicate at a minimum, and the results are expressed as mean ± standard deviation (SD). The data were analyzed by one-way ANOVA. Differences between groups were considered statistically significant if P < 0.05. Analyses were conducted using SPSS version 13.0 (Chicago, IL, USA).

## Results

### NaB inhibited proliferation and induced apoptosis in colorectal cancer cells

**Fig 1:**
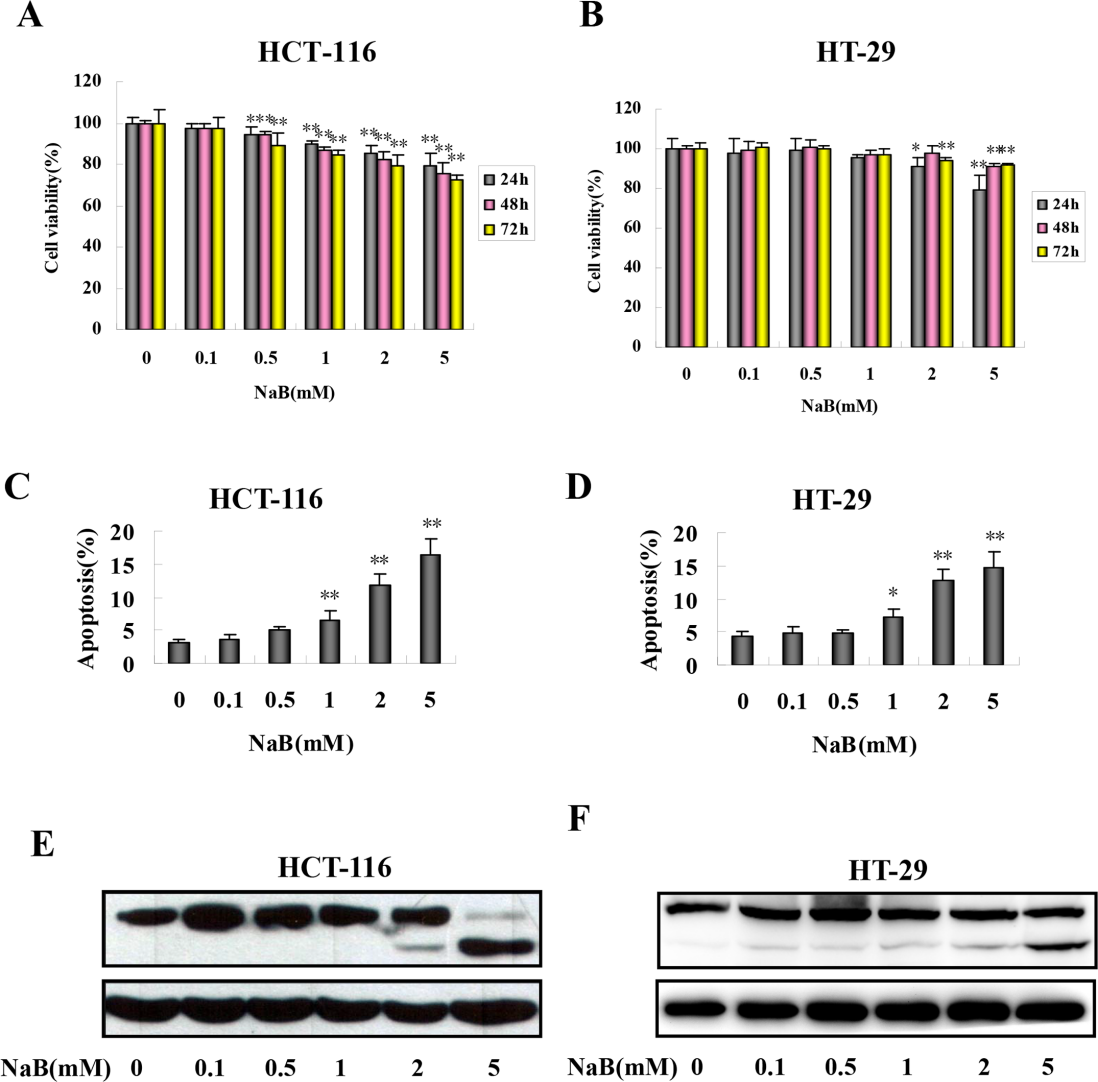
Sodium butyrate inhibited proliferation and induced apoptosis in colorectal cancer cells. HCT-116 (A) and HT-29 (B) cells were treated with the indicated concentrations of sodium butyrate (NaB) for 24 (grey bars), 48 (pink bars), or 72 (yellow bars) hours and cell proliferation was assessed. HCT-116 (C, E) and HT-29 (D, F) cells were treated with the indicated concentrations of NaB for 24 h. C.D. The percentage of apoptotic cells in HCT-116 (C) and HT-29 (D) cells was quantified in three independent experiments using an annexinV/PI assay (annexinV+/PI-) by flow cytometry and expressed as mean ± SD. One-way ANOVA was used to compare between the control cells and NaB treatments. *p<0.05, ** p<0.01 compared to control. E.F. Representative Western blots of full-length and cleaved PARP products in HCT-116 (E) and HT-29 (F) cells.

We first assessed the effects of NaB treatment on colorectal cancer cell proliferation and cell death. To assess the effect of NaB on proliferation, we quantified changes in MTT tetrazolium salt formation in response to NaB treatment in HCT-116 and HT-29 cells. NaB significantly reduced MTT tetrazolium salt formation in HCT-116 (Fig 1A) and HT-29 cells (Fig 1B) in a concentration- and time-dependent manner. In the HCT-116 cells, the lowest dose of NaB that significantly inhibited proliferation was 0.5mM, and the inhibition became apparent as soon as 24 h after treatment (Fig 1A). In the HT-29 cells, the lowest NaB dose that inhibited proliferation was 2mM, which again became apparent as soon as 24 h post treatment (Fig 1B). At the 2mM NaB dose, after 24 h the rate of proliferation relative to untreated control cells (100%) was approximately 85.10% in HCT-116 cells and 90.97% in HT-29 cells, respectively (Fig 1A and 1B). Given that a decrease in tetrazolium salt formation indicates fewer metabolically active cells, which could result from cell death or quiescence, we next quantified the extent of apoptosis in the cultures by flow cytometry using annexin V–FITC/PI staining 24 h after treatment with NaB. In both cell lines, NaB (1, 2, and 5 mM) significantly increased phosphotidylserine externalization (annexin V+; Fig 1C and 1D). The percentage of cells that were annexin V+ increased in a dose-dependent manner in both HCT-116 (Fig 1C) and HT-29 (Fig 1D) cells. The pro-apoptotic effect of NaB 24 h after treatment was corroborated by measuring PARP cleavage, a biochemical marker of apoptosis that is cleaved by Caspase-3, by Western-blot analysis. The expression of cleaved PARP was markedly increased at 2 and 5 mM NaB in the HCT-116 cells (Fig 1E) and HT-29 (Fig 1F) cells. This was consistent with the findings of the annexin V/PI assay (Fig 1C and 1D). Taken together, the results indicated that NaB treatment at the doses used inhibited CRC cell proliferation and induced apoptosis.

### NaB induced accumulation of acidic vesicular organelles and autolysosomes.

**Fig 2:**
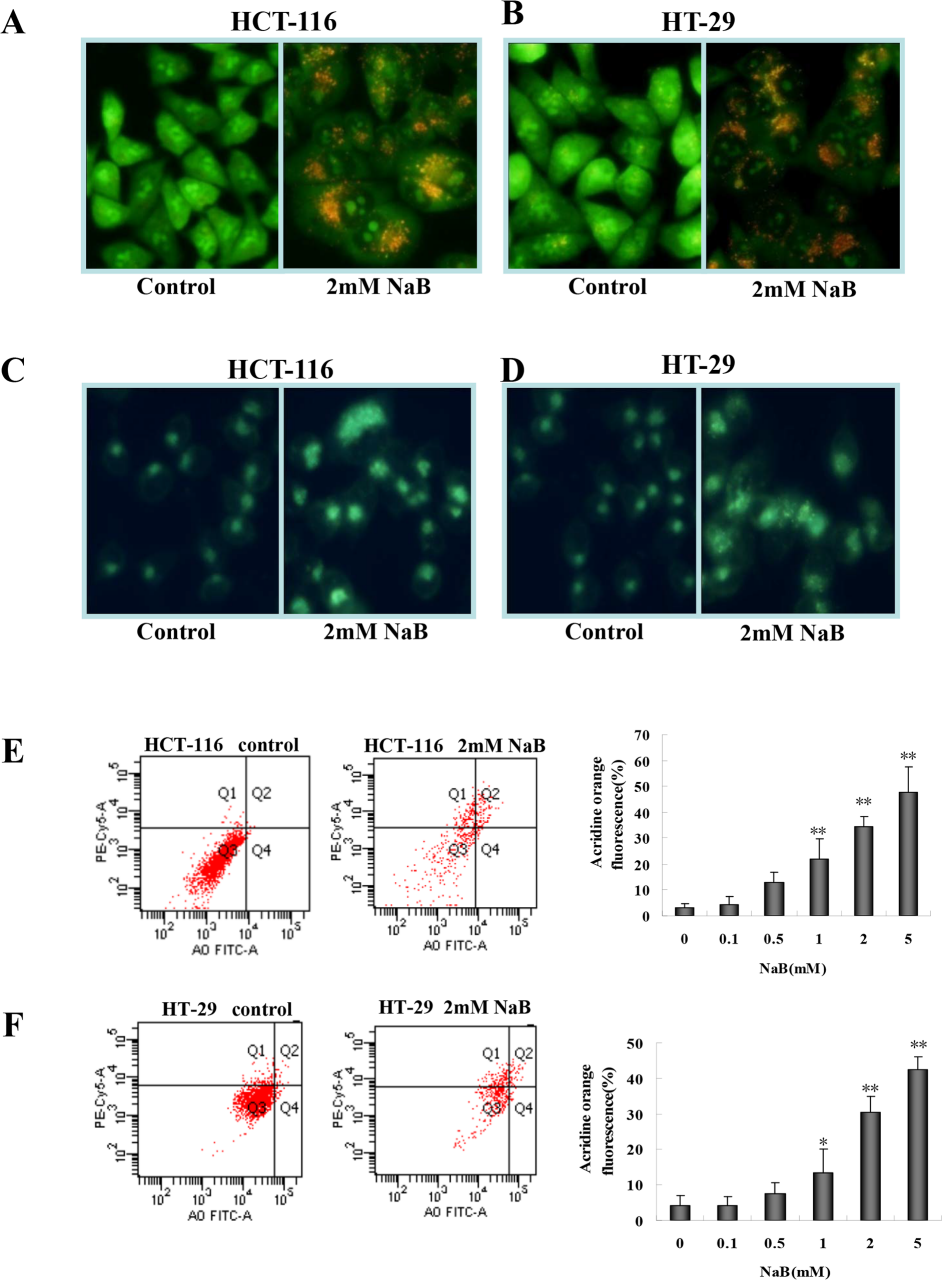
Sodium butyrate induced autophagy in colorectal cancer cells. HCT-116 (A, C) and HT-29 (B, D) cells were treated with 2mM sodium butyrate (NaB) for 24 h and then stained with acridine orange (AO; A-B) or monodansylcadaverine (MDC; C-D). Flow cytometry measured the percentage of AO+ cells and the results of three independent AO fluorescence experiments for HTC-116 (E) and HT-29 (F) cells were expressed as mean ± SD. One-way ANOVA was used to compare control cells and NaB treatments. *p<0.05, ** p<0.01 when compared to control.

Next, to assess whether NaB treatment as inducing autophagy we assessed the formation of acidic vesicular organelles and autolysosomes, both hallmarks of autophagy. We used acridine orange and monodansylcadaverine (MDC) staining to detect acidic vesicular organelles and autolysosomes, respectively [30]. Acridine orange emits dim red fluorescence in acidic vesicles, but is bright green in the cytoplasm and nucleus. NaB (2mM) enhanced the formation of acidic vesicular organelles (red foci in the cells) in HCT-116 (Fig 2A) and HT-29 (Fig 2B) cells compared to control cells (no apparent red staining). Similarly, NaB-treated HCT-116 (Fig 2C) and HT-29 (Fig 2D) cells stained with MDC showed a substantial increase in the number of fluorescent dots or vacuoles compared to control cells, indicating that NaB induced accumulation of autolysosomes. The accumulation of the acidic vesicles in acridine orange stained cells was quantified using a flow cytometry assay in HCT-116 (Fig 2E) and HT-29 (Fig 2F) cells. In both the HCT-116 and HT-29 cells, there was a significant dose-dependent increase in the percentage of fluorescent cells 24 h after NaB treatment (2 and 5mM), indicating an increase in the number of autophagic cells. At the 2mM NaB dose approximately 38.3% of the HCT-116 cells and 34.6% of the HT-29 cells, respectively appeared to be autophagic (Fig 2E and 2F).

### NaB induced autophagy in colorectal cancer cells

**Fig 3:**
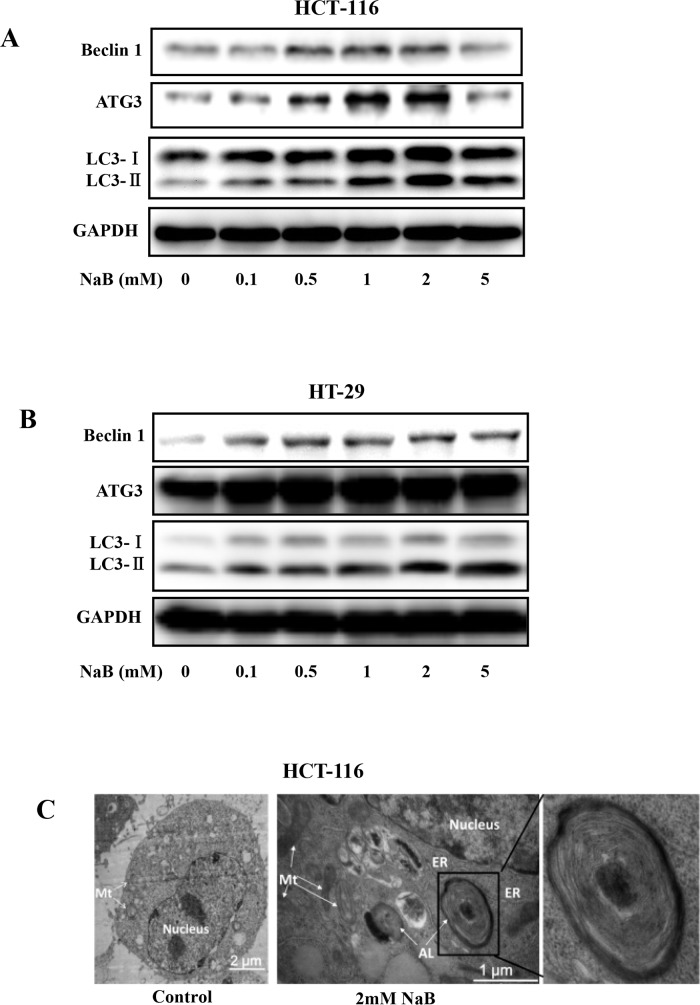
Sodium butyrate increased expression of LC3-II. HCT-116 (A) or HT-29 (B) cells were treated with the indicated concentrations of sodium butyrate (NaB) for 24 h. Representative blots showed expression of Beclin1, ATG3, and LC3 as well as GAPDH, the loading control. C. Electron microscopy images of control and NaB (2mM) treated HCT-116 cells. ER, endoplasmic reticulum; Mt, mitochondrion; AL.

To further verify that NaB treatment was inducing autophagy in the CRC cell lines, the conversion of free LC3-I to lipid bound LC3-II was assessed. LC3 is an important constituent of the autophagosome and widely used as a biochemical marker of autophagy [31]. A marked conversion of free LC3-I to heavier lipid bound LC3-II was detected after exposing HCT-116 (Fig 3A) and HT-29 (Fig 3B) cells to 2mM sodium butyrate for 24 h. The accumulation of LC3-II was positively correlated with the treatment dose, suggesting that NaB induced autophagy in a dose-dependent manner. Surprisingly, HCT-116 cells treated with 5mM NaB was the only condition in which LC3-II levels were reduced compared to lower NaB doses (Fig 3A). NaB also induced autophagy in a time-dependent manner (data not shown). We further verified the presence of autophagy by measuring levels of ATG3, an essential regulatory component of autophagosome biogenesis [32], and Beclin-1 (Atg 6), which has a central role in autophagy. Beclin-1 and its binding partner PI3K, are required to initiate formation of the autophagosome [33]. NaB treatment increased levels of both ATG3 and Beclin1 in a dose dependent manner (Fig 3A and 3B). Likewise, mRNA levels of Beclin 1 and LC3B, but not ATG3, significantly increased with increasing doses of NaBu in a dose dependent manner (S1 Fig) Autophagy induction was further confirmed by electron microscopy. The accumulation of double membrane vesicles in HCT-116 cells treated with 2mM NaB for 24 h clearly indicated autophagosome formation (Fig 3C, right panel).

### Effects of 3-methyladenine and chloroquine on NaB-induced autophagy in colorectal cells

**Fig 4:**
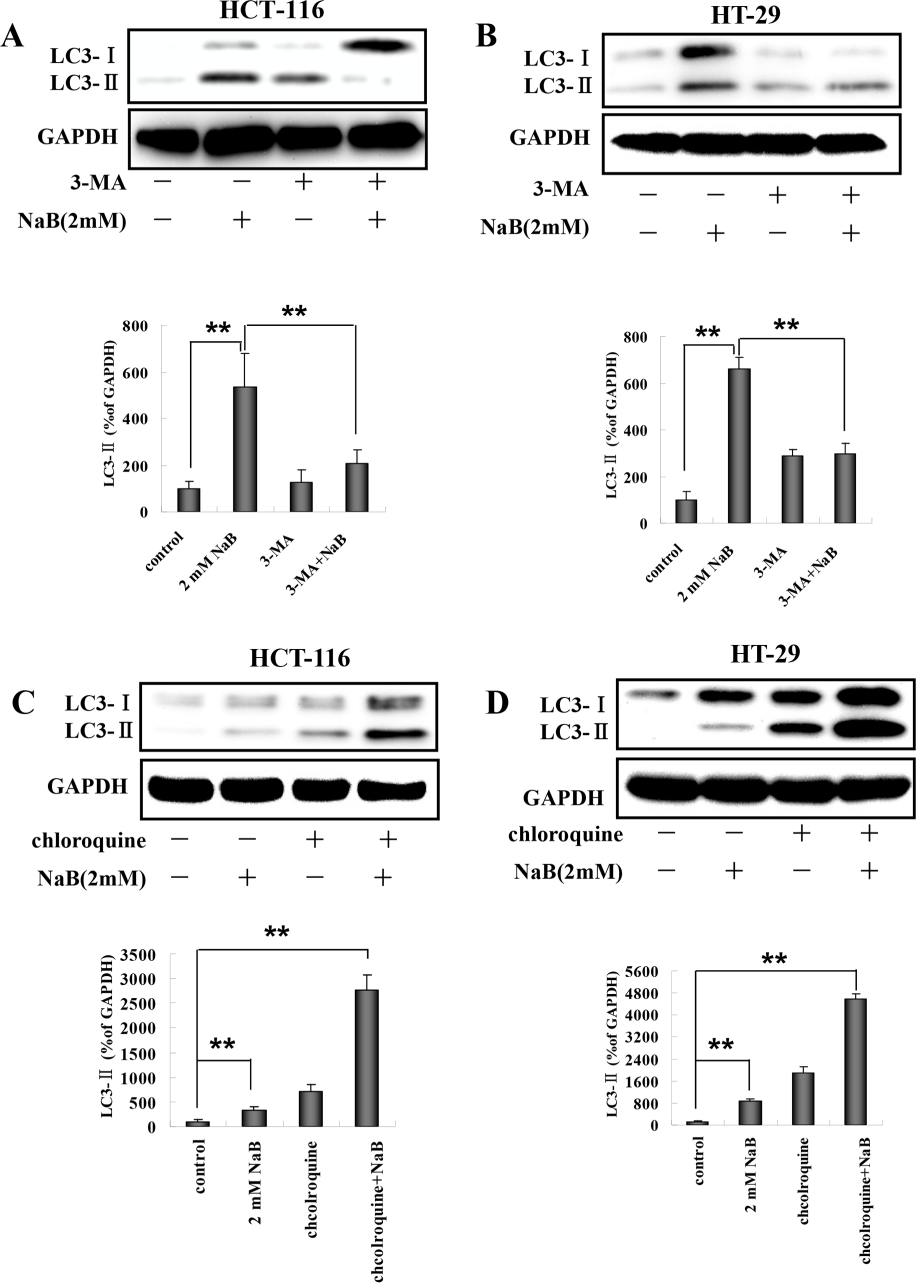
3-MA and chloroquine blocked sodium butyrate induced autophagy in colorectal cancer cells. HCT-116 or HT-29 cells were treated with 5mM 3-MA or 5μM chloroquine (CQ) for 30 min, and then with 2mM sodium butyrate (NaB) for 24 h. Representative Western blots of LC3-I and LC3-II expression were quantified by densitometry and normalized to GAPDH (ratio of LC3:GAPDH). The fold change from control is indicated as the mean ± SD of three independent experiments. One-way ANOVA was used for statistical analysis. ** p<0.01, compared to the respective control group. (A) HCT-116 cells treated with 3-MA and NaB; (B) HT-29 cells treated with 3-MA and NaB; (C) HCT-116 cells treated with CQ and NaB; (D) HT-20 cells treated with CQ and NaB.

We next tested whether pretreatment with 3-methyladenine (3-MA), a compound which inhibits PI3K, could prevent the NaB-induced conversion of LC3-I to LC3-II. Pretreatment with 3-MA significantly reduced the level of LC3-II induced by NaB compared to NaB treated cells without 3-MA in HCT-116 (Fig 4A) and HT-29 (Fig 4B) cells. LC3-II is degraded in the lysosomes during autophagy, however, it can accumulate if protein degradation is inhibited. To more precisely determine whether the level of LC3-II was increased due to autophagy, we assessed LC3-II accumulation in the presence of chloroquine, which blocks degradation of autophagosome contents. HCT-116 (Fig 4C) and HT-29 (Fig 4D) cells were exposed to chloroquine for 30 min and then treated with NaB for 24 h. As expected, chloroquine treatment alone increased the accumulation of LC3-II, indicating we successfully blocked LC3 degradation. The levels of LC3-II when chloroquine and NaB were combined were higher than LC3-II expression induced by NaB alone. These findings provided additional confirmation that autophagic flux was increased in presence of NaB in our model.

### NaB induced ER stress in colorectal cancer cells

**Fig 5:**
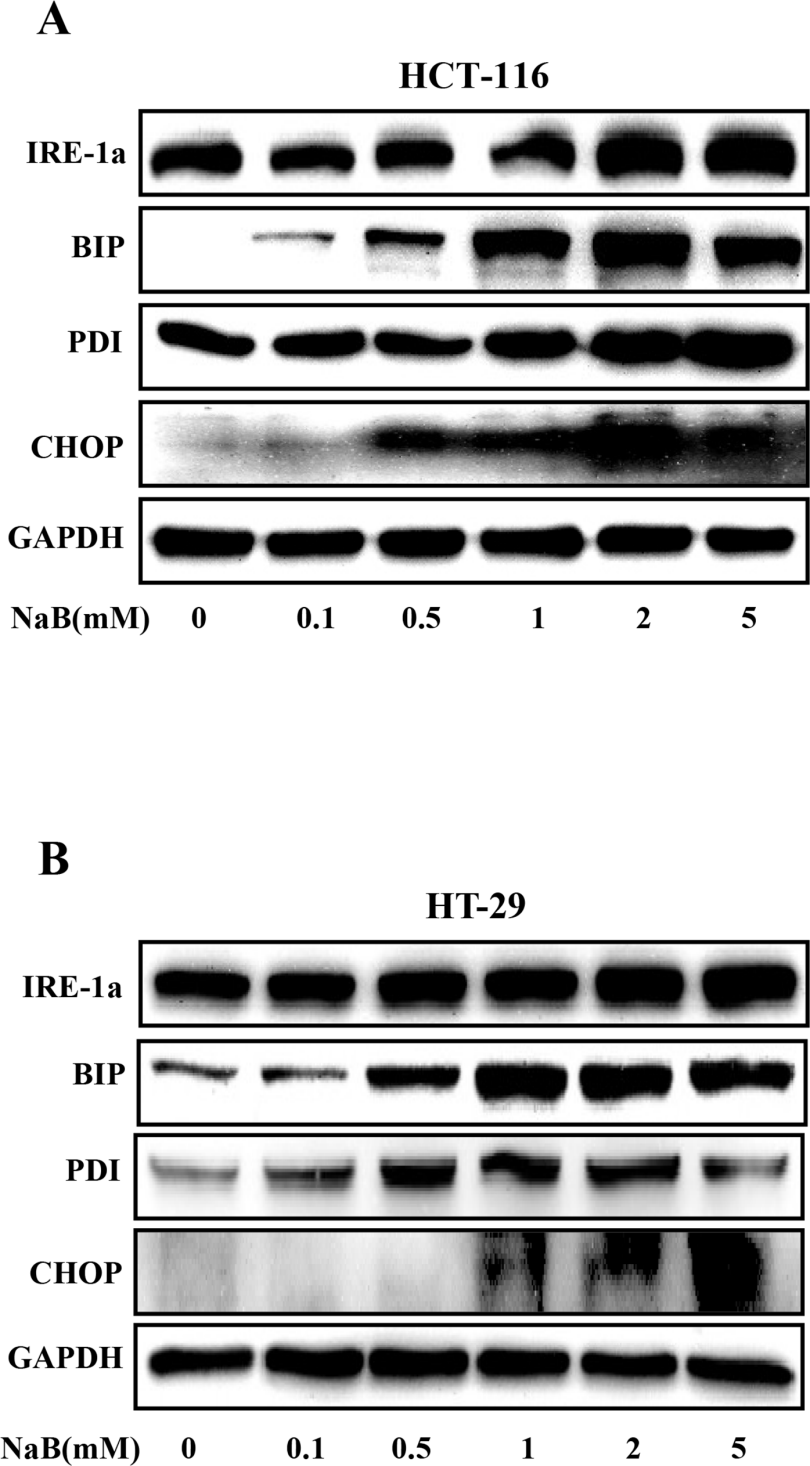
Sodium butyrate induced endoplasmic reticulum stress in colorectal cancer cells. HCT-116 (A) and HT-29 (B) cells were treated with the indicated concentrations of sodium butyrate (NaB) for 24 h. Representative Western blots show the expression of IRE-1a, BIP, PDI, and CHOP. GAPDH was used as the loading control.

Previous studies show that disruptions in the integrity of the ER activate autophagy [24–26]. Therefore, we hypothesized that NaB might cause ER stress in CRC cells. To address this, HCT-116 and HT-29 cells were treated with 0–5mM NaB, and then Western blot was used to probe for markers of ER stress. Our results demonstrated that NaB substantially increased the expression of binding immunoglobulin protein (BIP) in a concentration dependent manner in HCT-116 and HT-29 cells (Fig 5A and 5B), indicating ER stress. BIP regulates the expression of inositol-requiring enzyme 1a (IRE1a), C/EBP homologous protein (CHOP), and protein disulfide isomerase (PDI) during ER stress; and consistent with increased BIP, the levels of CHOP, PDI, and IRE1a were also elevated (Fig 5A and 5B), providing further support for the hypothesis that NaB induced ER stress in CRC cells.

### Effects of cycloheximide and mithramycin on NaB-induced ER stress in colorectal cells

**Fig 6:**
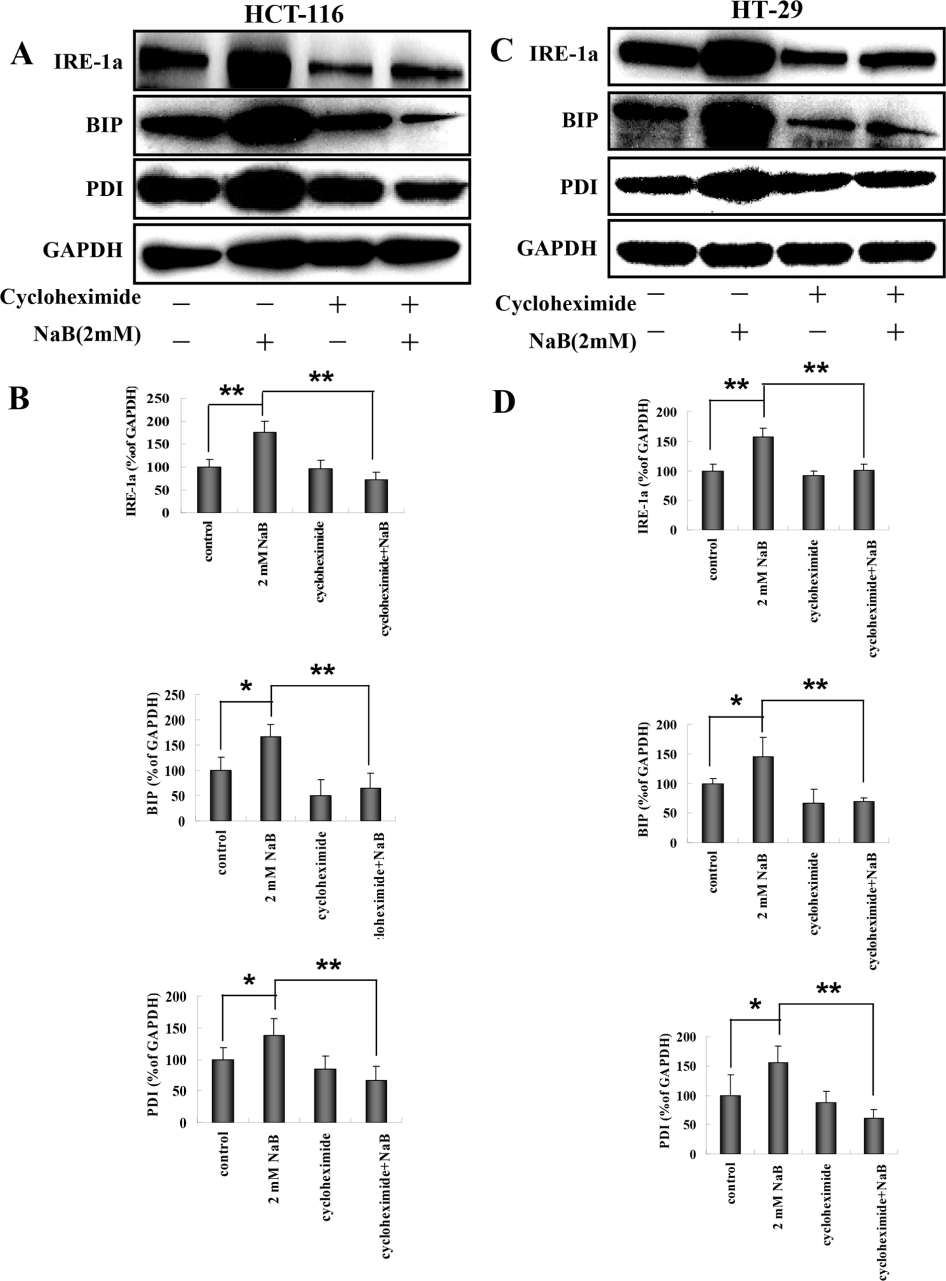
Cycloheximide blocked sodium butyrate induced endoplasmic reticulum stress in colorectal cancer cells. HCT-116 or HT-29 cells were treated with 10 μg/mL cycloheximide for 30 min followed by sodium butyrate (NaB) for 24 h. Representative Western blots showed the expression of IRE-1a, BIP, PDI, and GADPH (loading control) in HCT-116 (A) or HT-29 (C) cells. Protein expression was quantified by densitometry and normalized to GAPDH (ratio of protein:GAPDH). The fold change from control for each protein is expressed as mean ± SD of three independent experiments for HCT-116 (B) and HT-29 (D) cells. are shown. One-way ANOVA was used for statistical analysis. * P<0.05, ** p<0.01, compared to the respective control group.

The increased levels of BIP, PDI, and IRE1a that were observed following treatment with NaB are indicative of an unfolded protein response (UPR), therefore we investigated whether blocking protein synthesis using cyclohexamide (CHX) inhibited expression of BIP, PDI, and IRE1a in NaB treated cells. HCT-116 and HT-29 cells were treated with 10 μg/mL CHX for 1 h and then treated with 2mM NaB for 24 h. Fig 6A shows a representative Western blot from HCT-116 cells. The expression levels of IRE1a, BIP, and PD1 were quantified using densitometry and normalized to GAPDH levels (Fig 6B). Treatment with NaB significantly increased the levels of IRE1a, BIP, and PDI compared to control cells, as expected. Blocking protein synthesis using CHX abrogated the effects of NaB in HCT-116 cells and the levels of IRE1a, BIP, and PDI were significantly reduced compared to NaB treated cells (Fig 6B). Interestingly, the expression levels of IRE1a, BIP, and PDI were also lower than control cells in the presence of CHX + NaB. Similar results were obtained using HT-29 cells (Fig 6C and 6D).

**Fig 7:**
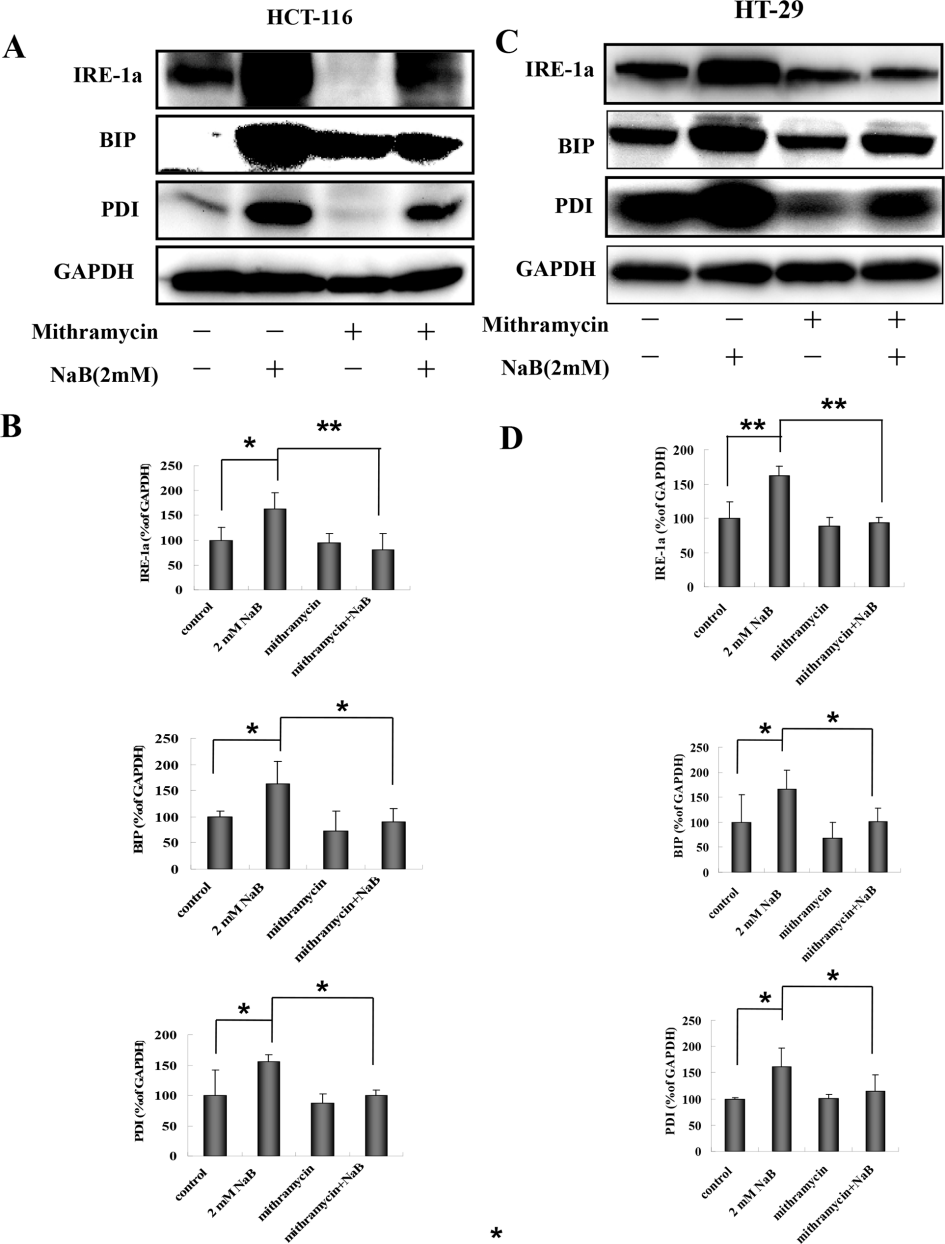
Mithramycin blocked sodium butyrate induced endoplasmic reticulum stress in colorectal cancer cells. HCT-116 (A, B) or HT-29 (C,D) cells were treated with 0.1μM mithramycin for 30 min followed by sodium butyrate (NaB) for 24 h. Representative Western blots showing the expression of IRE-1a, BIP, and PDI in HCT-116 (A) or HT-29 (C) cells are shown. GAPDH was used as loading control. Protein expression (IRE-1a, BIP, and PDI) was quantified by densitometry and normalized to GAPDH (ratio of protein:GAPDH). The fold change from control for each protein is expressed as mean ± SD of three independent experiments for HCT-116 (B) and HT-29 (D) cells. One-way ANOVA was used for statistical analysis. * P<0.05, ** p<0.01, compared to the respective control group.

CHX is a general protein synthesis inhibitor, therefore to confirm that ER stress specifically was induced in our model, we used mithramycin, a specific ER stress inhibitor. Mithramycin is a gene selective sp1 inhibitor that blocks transcription and protein synthesis of ER chaperones [34]. The cells were treated with 0.1μM mithramycin for 30 min and then with 2mM NaB for 24 h. A representative Western blot showing IRE1a, BIP, and PDI levels post treatment in HCT-116 cells is shown in Fig 7A. The protein expression levels were quantified for statistical comparison (Fig 7B). Similar to the CHX results, mythramicin + NaB significantly reduced the levels of all three markers compared to NaB treated cells alone (Fig 7B). Similar results were obtained using HT-29 cells (Fig 7C and 7D). These results strongly support that NaB activated ER stress in colorectal cancer cells.

### Blocking ER stress inhibited NaB-induced autophagy in colorectal cells

**Fig 8:**
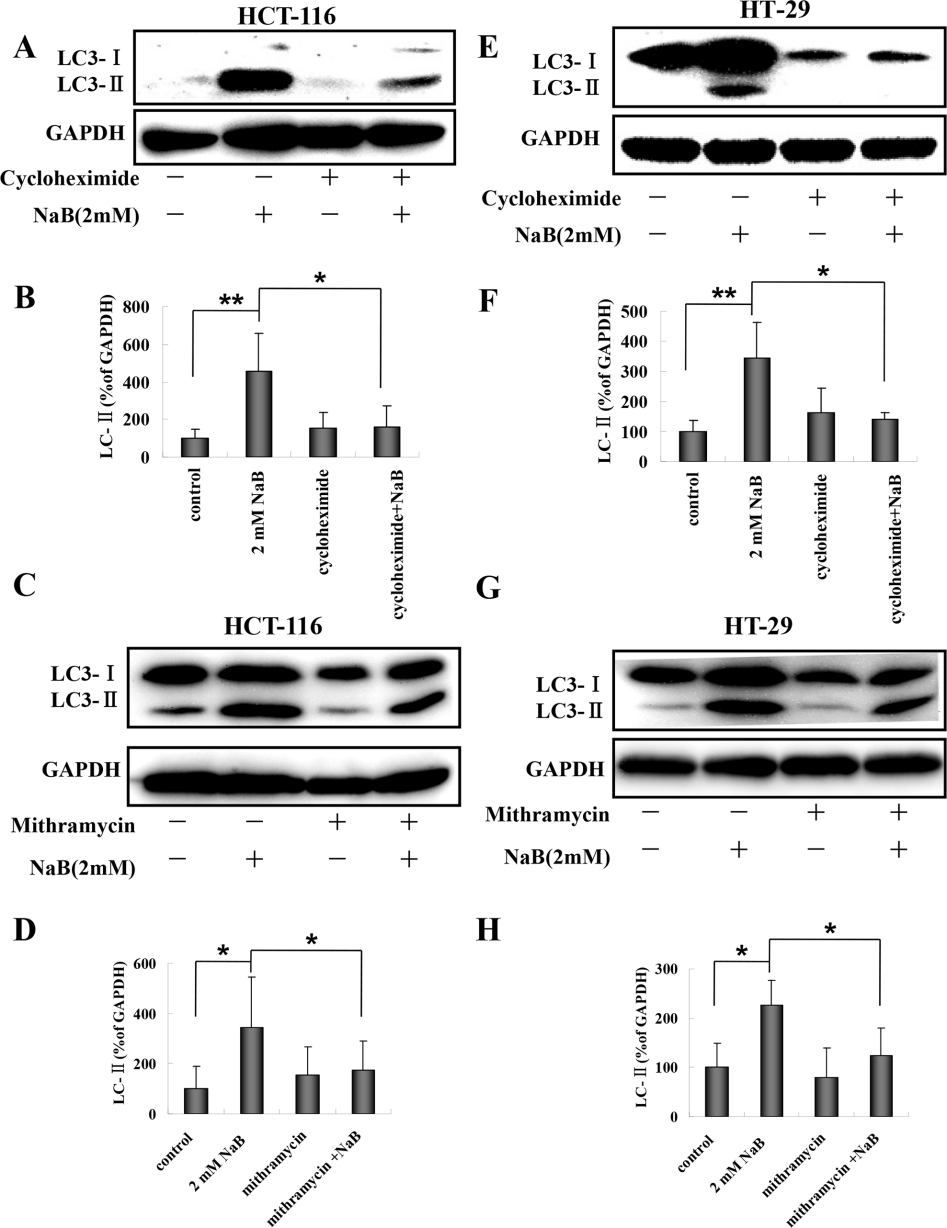
Cyclohexamide and mithramycin blocked sodium butyrate induced autophagy in colorectal cancer cells. HCT-116 or HT-29 cells were treated with 10 μg/mL cycloheximide or 0.1μM mithramycin for 30 min and then with 2mM sodium butyrate (NaB) for 24 h. Representative Western blots of the expression of LC3-II are shown. The level of LC3-II expression was quantified by densitometry and normalized to GAPDH (ratio of LC3-II:GAPDH). The fold change from control cells is shown. Means and standard deviation (SD) of three independent experiments are shown. One-way ANOVA was used for statistical analysis. * P<0.05, ** p<0.01, compared to the control group. (A-B) HCT-116 cells treated with cyclohexamide; (C-D) HCT-116 cells treated with mithramycin; (E-F) HT-29 cells treated with cyclohexamide; (G-H) HT-29 cells treated with mithramycin.

A number of studies have suggested that disturbances in ER homeostasis lead to autophagy [21, 25, 26]. Having established that NaB treatment is associated with ER stress markers (IRE1a, BIP, and PDI) and that NaB treated cells undergo autophagy, we next assessed whether NaB-induced ER stress led to the formation of autophagic vesicles by measuring membrane bound LC3-II in NaB treated HCT-116 and HT-29 cells pretreated with mithramycin or CHX. A representative Western blot showing LC3-I and LC3-II levels in HCT-116 cells is shown in Fig 8A. Treatment with NaB significantly increased the levels of LC3-II as expected. Pretreatment with CHX significantly reduced the levels of LC3-II in NaB treated cells compared to NaB treated cells without CHX (Fig 8B). Similar results were obtained for mithramycin in HCT-116 cells (Fig 8C and 8D). HT-29 cell also had significantly reduced levels of LC3-II following pretreatment with CHX (Fig 8E and 8F) or mithramycin (Fig 8G and 8H) compared to NaB treatment in the absence of protein synthesis inhibitors. These results support our hypothesis that ER stress was involved in the regulation of NaB-mediated autophagy in colorectal cancer cells.

**Fig 9:**
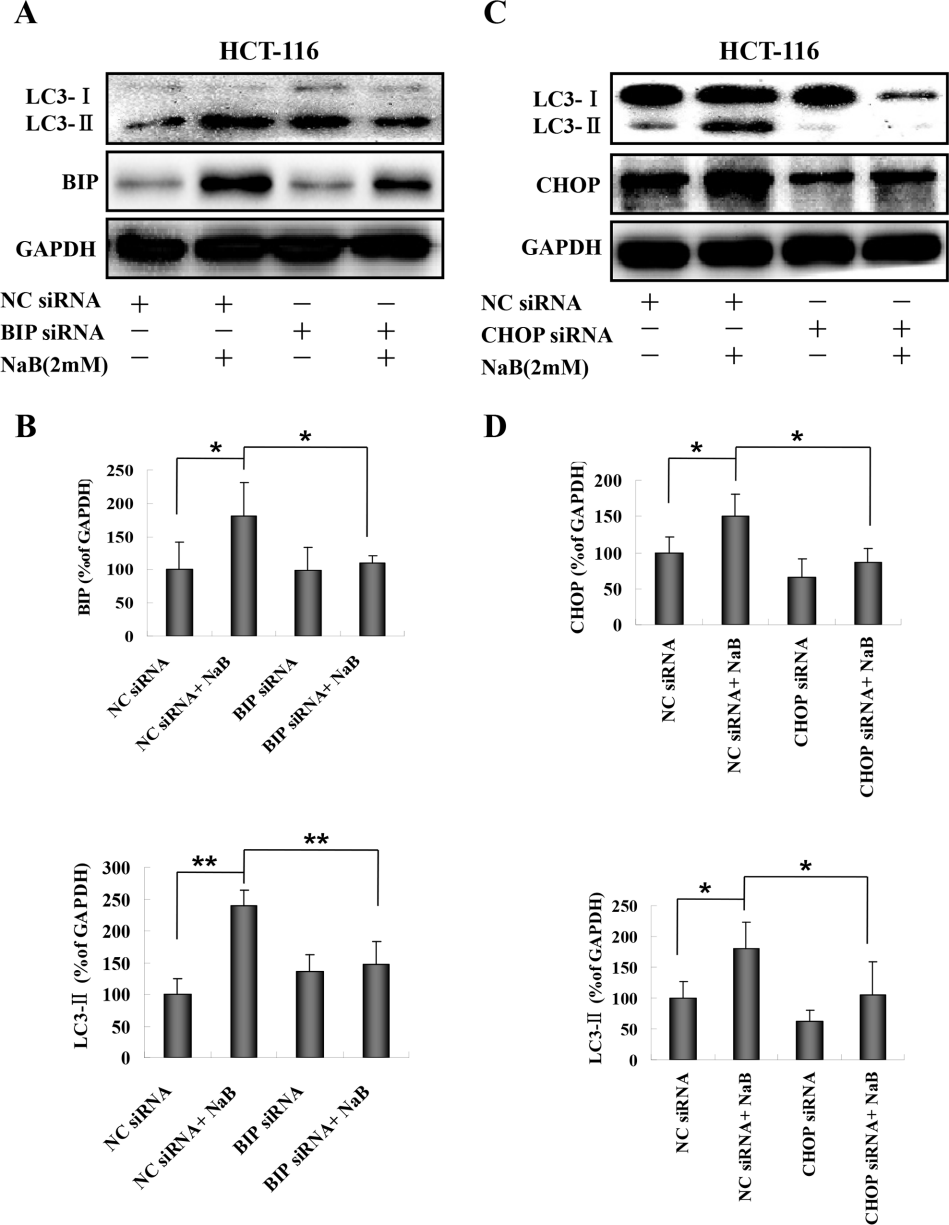
RNA interference targeting BIP or CHOP blocked sodium butyrate induced autophagy in colorectal cancer cells. HCT-116 cells were transfected with BIP or CHOP specific siRNAs for 48 h and then treated with or without 2mM sodium butyrate (NaB) for 24 h. Negative control (NC) scramble siRNA was used the negative control for the transfection. Representative Western blots are shown for BIP siRNA (A) and CHOP siRNA (C). The expression level of each protein was determined by densitometry and normalized to GAPDH (ratio of protein:GAPDH). (B) Normalized expression levels of BIP and LC3-II in HCT-116 cells treated with BIP specific siRNA. (C) Normalized expression levels of CHOP and LC3-II in HCT-116 cells treated with CHOP specific siRNA. Means and standard deviation (SD) of three independent experiments are shown. One-way ANOVA was used for statistical analysis. * P<0.05, ** p<0.01, compared to the respective control group.

Both mithramycin and CHX treatment blocked induction of molecules that regulate ER stress and autophagy. However, chemical inhibitors are known to be associated with off-target effects, therefore we confirmed our results regarding the involvement of BIP and CHOP in NaB-induced autophagy using RNA interference. HCT-116 and HT-29 cells (HT-29 data not shown) were transfected with specific siRNAs targeting BIP or CHOP and then treated with NaB. Cells transfected with scrambled siRNA were used as the negative control. A representative Western blot showing LC3-II and BIP expression is shown in Fig 9A. The protein levels were quantified by densitometry (Fig 9B). While there was no effect of siRNA treatment on basal levels of BIP or LC3-II in HCT-116 cells, in the NaB treated cells BIP-specific siRNA prevented the expected increase in LC3-II and in most experiments also the rise in BIP levels (Fig 9B). CHOP-specific siRNA treatment also prevented the expected increase in LC3-II and in most experiments also the rise in CHOP levels (Fig 9C and 9D). These results strongly suggested that NaB induced autophagy was mediated by ER stress in CRC cells.

### Inhibition of autophagy enhanced NaB-induced apoptotic cell death

**Fig 10:**
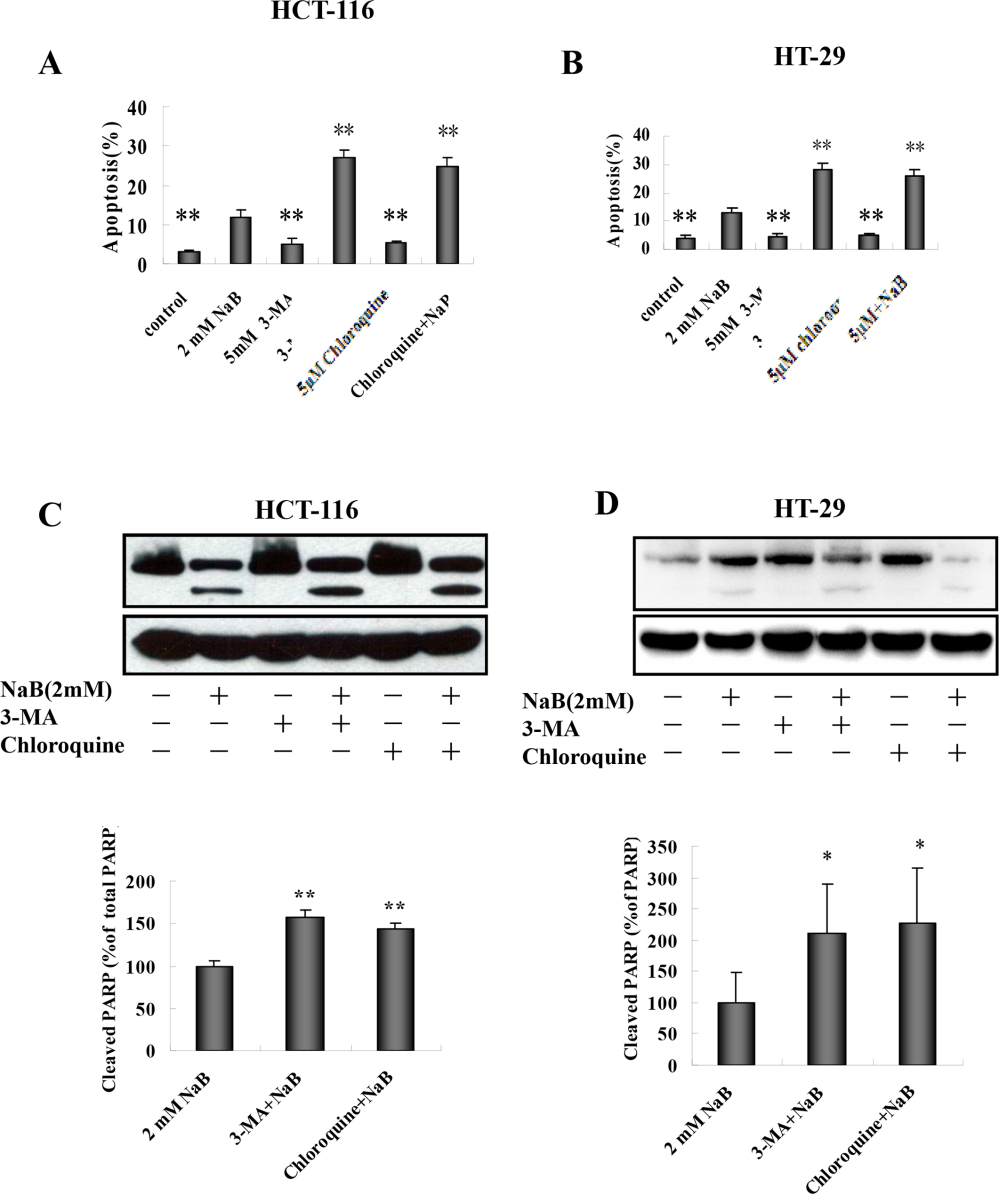
Inhibition of autophagy potentiates sodium butyrate-induced apoptotic cell death. HCT-116 (A) and HT-29 (B) cells were exposed to 5mM 3-MA or 5μM chloroquine (CQ) for 30 min and then treated with 2mM sodium butyrate (NaB) for 24 h. The percentage of apoptotic cells (annexinV+/PI-) was quantified by flow cytometry using an annexinV/PI assay and expressed as mean ± SD of three independent experiments. One-way ANOVA was used for statistical analysis to compare control cells and NaB treatments. *p<0.05, ** p<0.01 compared to control. (C.D.) Cells were treated as described above and the proteins were harvested for Western blot analysis. Expression of full-length and cleaved PARP in HCT-116 (C) or HT-29 (D) cells was compared to GAPDH, the loading control. The ratio of cleaved PARP to total PARP was calculated and the fold change in normalized PARP expression relative to cells treated with 2mM NaB is shown as the mean ± SD of three independent experiments. One-way ANOVA was used for statistical analysis. *P<0.05, ** p<0.01, compared to the 2mM NaB group.

Crosstalk between autophagy and apoptosis is widely accepted [35]. To explore the connection between NaB-induced autophagy and apoptosis, the effects of 3-MA and chloroquine on apoptosis in HCT-116 and HT-29 cells treated with NaB were examined. Cells were pretreated with 3-Ma (5mM) or chloroquine (5μM) for 30 min and then treated with NaB (2mM) for 24 h. Apoptosis was detected using annexinV/PI staining. Both 3-MA and chloroquine treatment significantly enhanced the cytotoxic effect of NaB in HCT-116 cells (Fig 10A, S2 Fig) and HT-29 cells (Fig 10B, S2 Fig). As an independent approach to assessing cancer cell apoptosis, PARP cleavage was assessed by Western blot. Similar to the flow cytometry results, 3-MA and chloroquine significantly increased the levels of cleaved PARP in HCT-116 (Fig 10C) and HT-29 (Fig 10D) cells. Taken together, these results suggested that the autophagic response protected CRC cells from NaB-induced apoptosis.

## Discussion

The effects of butyrate, and indirectly the utility of increasing dietary fiber, in preventing and treating CRC remain unclear. Here, we show that NaB induced both autophagy and apoptosis in human CRC cells in vitro. This observation is in agreement with previous reports from several groups [36, 37]. We concluded that CRC cells were undergoing autophagy based on increased expression of LC3-II, ATG3, and beclin 1. The accumulation of LC3-II and its localization to vesicular structures are commonly used as markers of autophagy [22]. ATG3 is an essential regulatory component of autophagosome biogenesis [32]. Beclin-1 and its binding partner PI3K are required for the initiation of the formation of the autophagosome in autophagy [33]. In addition, 3-MA and chloroquine, common autophagy inhibitors, attenuated the NaB-induced increase in LC3-II in CRC cells, consistent with previous studies [38, 39]. Finally, we found that inhibiting autophagy increased the level of apoptosis in CRC cells, suggesting that autophagy was acting as a protective pathway in human CRC cells.

Previously, we demonstrated that sodium butyrate induced calcium release from the ER, which caused an extracellular calcium influx in colorectal cancer cells [27] ER stress is known to be one of the major inducers of autophagy [25, 26]. Here we show that NaB-induced autophagy was mediated by activation of ER stress, which was associated with an increase in the expression of BIP, IRE1a, CHOP, and PDI. BIP is a protein folding chaperone. Under conditions of ER stress, misfolded proteins accumulate in the ER lumen, and BIP dissociates from IRE1a, allowing its activation, which leads to expression of the transcriptional factor CHOP [40]. Changes in PDI activity are associated with protein misfolding and ER stress [41]. We were able to attenuate autophagy by inhibiting the ER stress response pharmacologically (cycloheximide and mithramycin) or genetically (BIP and CHOP siRNA), and observed a simultaneous reduction in autophagy. Similar to our observations, autophagy associated with ER stress has been observed after treatment with atorvastatin and palmitate in other malignancies [42, 43].

Autophagy is a primarily pro-survival pathway that cells undergo during nutrient depletion, starvation, or hypoxia [44]. However, excessive or prolonged autophagy can also lead to cell death [45]. Thus, autophagy is often referred to as a double-edged sword [46] Tang et al. were the first to demonstrate that SCFAs induce autophagy in human CRC cells to dampen apoptosis, but that inhibition of autophagy potentiated SCFA-induced apoptosis [47]. In our study, we observed a potential dose related response wherein, butyrate at low doses (2mM) induced autophagy in HCT-116 cells, but at high doses (5mM) the levels of autophagy appeared to be reduced. However, apoptosis was clearly induced at high levels in response to the 5mM dose of butyrate. Autophagy induction is consistent with the results published by Tang et al. indicating autophagy occurred when HCT-116 cells were treated with 1–3mM butyrate [10]. However, in contrast to our studies, Tang et al. reported autophagy induction in the absence of overt apoptosis at this concentration of butyrate in HCT-116 cells [10]. The reason for the apparent difference in study results is not clear. We also observed subtle differences in the responses of the HCT-116 and HT-29 cells to NaB treatment. For example, cell proliferation was inhibited at a much lower dose in the HCT-116 cells (0.5mM NaB) than in the HT-29 cells (2mM), and the HT-29 cells did not have the same apparent reduction in autophagy (Atg3 accumulation) as the HCT-116 cells at the 5mM dose of NaB. Bordonaro et al. have proposed that the different effects of butyrate in CRC cells and normal colonocytes may be determined in part by canonical Wnt signaling thresholds; moderately elevated Wnt activity is a key component of CRC tumorigenesis however, when butyrate triggers additional Wnt signaling CRC cells become sensitized to undergo apoptosis. In contrast, in normal colonocytes with low basal levels of Wnt signaling butyrate-mediated increases in Wnt signaling lead to proliferation and growth [48]. A previous study reported that HCT-116 cells have a relatively high level of Wnt signaling in response to NaB while HT-29 cells have relatively low levels of Wnt activation in response to NaB. The same report documented a linear relationship between the level of Wnt activation and apoptosis in CRC cell lines treated with NaB (5mM, 24 h)[49]. Thus, it is possible that subtle differences in intracellular signaling thresholds between the HCT-116 and HT-29 cells may have contributed to the differences we observed in our study, suggesting that different CRC subtypes might respond to butyrate therapy differently in clinical setting.

Autophagy has been shown to interact with apoptosis in three ways: (1) as an inhibitor of apoptosis; (2) as a facilitator of apoptosis; and (3) cooperatively with apoptosis to trigger cell death [35]. In some models, autophagy has been proposed as an alternative to necrotic cell death in apoptosis deficient tumors to limit inflammation and tumorigenesis [50]. The BCL-2 protein family members have been proposed as dual regulators of apoptosis and autophagy. During apoptosis, the BCL-2 family members can have either pro-apoptotic (Bax, Bak, and Bok) or anti-apoptotic functions (BCL-2, BCL-XL, and BCL-W) [35].The induction of apoptosis is associated with relocation of the cytosolic pro-apoptotic BCL-2 family proteins to the mitochondria where they trigger release of cytochrome C and apoptosis. Pro-apoptotic BCL-2 family members such as Bak also localize to the ER. BCL-2 can directly inhibit autophagy by binding to the beclin 1/PI3K complex, however, under conditions of stress BCL-2 is phosphorylated and inhibited, releasing the beclin 1/PI3K complex and allowing autophagy to occur [35]. Thus the molecular mechanisms that determine the autophagic or apoptotic response to NaB at low and high doses, respectively, remain to be determined.

Autophagy has been shown to have a complex relationship with tumor(s). Autophagy is induced in response to chemotoxic agents such as camptothecan (breast cancer) and 5-fluorouracil (5-FU; colon and esophageal cells), and may contribute to developing drug resistance by allowing tumor cells to persist [19]. A search of the clinicaltrials.gov database (registered US clinical trials) returns 49 results for studies investigating autophagy and cancer. Of these, nearly half are using hydroxychloroquine or chloroquine to inhibit autophagy in conjunction with an established cancer drug (e.g. sirolimus, 5-FU). Given that NaB treatment induces both apoptosis and autophagy, it is likely that NaB would have its optimal effects (most anti-tumorigenic) when paired with an autophagy inhibitor in vivo.

While we were able to show that NaB treatment increases the levels of both autophagy and apoptosis in vitro, the effects remain to be confirmed in vivo in robust animal models. Any studies using butyrate as an anti-cancer drug will require careful dose titration studies in vivo, as the effects of butyrate treatment at low doses may be limited since the endogenous butyrate levels reach millimolar (mM) levels in the colon. In our work, we saw limited evidence that NaB may induce autophagy at low doses (2mM) and apoptosis at higher doses (5mM). While this observation remains to be confirmed, it may be relevant when taking into account the endogenous butyrate concentration and exogenous NaB treatment in patients. Furthermore, the sensitivity of human primary CRC cells to multiple NaB concentrations in vitro also warrants investigation. Finally, the molecular mechanism of NaB activity remains to be determined. SCFA-induced autophagy is associated with cellular ATP depletion and ROS generation, both of which contribute to AMPK activation and consequential mTOR inhibition [10, 47]. It is possible that ER stress may engage in cross-talk with the AMPK/mTor pathway. Better understanding the molecular mechanism of action for NaB may help inform future targeted drug development efforts.

In summary, we have demonstrated that NaB-induced autophagy in cultured CRC cells by activating ER stress. Autophagy was likely increased as a protective response against NaB-induced cell apoptosis. These findings are consistent with others that suggest regulating autophagy could be a therapeutic approach in CRC. Importantly, we present evidence showing the involvement of ER stress in NaB-induced autophagy, which provides support for investigating the interaction of the UPR and butyric acid to suppress carcinogenesis in the colon.

## Supporting Information

S1 Fig mRNA expression of Bectin1, ATG3, and LC3B by real-time PCR. After appropriate treatments to HCT-116 and HT-29 cells in 6-well plates, RNA was extracted by using TRIzol (Invitrogen,15596–026), and 500ng of RNA was reverse-transcribed to cDNA. Beclin1, ATG3 and LC3B mRNA levels were determined by using PrimeScript RT reagent Kit (Perfect Real Time) (TAKARA, RR037A). Beclin1 and LC3B, but not ATG3, mRNA levels significantly increased in comparison to controls.
(PPT)

S2 Fig Mithramycin pretreatment reduced NaBu effect on PARP cleavage and apoptosis. **(A)** Representative Western blots showed the PARP expression in HCT-116 or HT-29 cells that were exposed to 0.1μM Mithramycin for 30 minutes followed by treatment with 2mM NaB for 24 hours. GAPDH was used as loading control. **(B, C)** HCT-116 (B) or HT-29 (C) cells were exposed to 0.1μM Mithramycin for 30 minutes followed by treatment with 2mM NaB for 24 hours in three independent experiments. Flow cytometry showed the percentage of annexin-5/PI (apoptotic cells), which was expressed as the mean ± SD of three independent experiments. One-way ANOVA was used for statistical analysis to compare control cells and NaB treatments. *p<0.05, ** p<0.01 compared to control.
(PPT)

S1 Table Primer sequences for quantitative real-time PCR. PCR amplified products were detected using SYBR^®^ Premix Ex Taq^™^ II (Tli RNaseH Plus) (TAKARA, RR820A). Consistent amplification of DNA was detected by fluorescence of SYBR Green I in real time PCR.
(DOC)
