# Supplementary material for: Sodium Butyrate Induces Endoplasmic Reticulum Stress and Autophagy in Colorectal Cells: Implications for Apoptosis
Source: PLoS One. 2016 Jan 19;11(1):e0147218. doi: 10.1371/journal.pone.0147218 (PMC4718706; doi:10.1371/journal.pone.0147218)
Supplement: S1 Fig — After appropriate treatments to HCT-116 and HT-29 cells in 6-well plates, RNA was extracted by using TRIzol (Invitrogen,15596–026), and 500ng of RNA was reverse-transcribed to cDNA. Beclin1, ATG3 and LC3B mRNA levels were determined by using PrimeScript RT reagent Kit (Perfect Real Time) (TAKARA, RR037A). Beclin1 and LC3B, but not ATG3, mRNA levels significantly increased in comparison to controls. (PPT) [file pone.0147218.s001.ppt]

## Slide 1
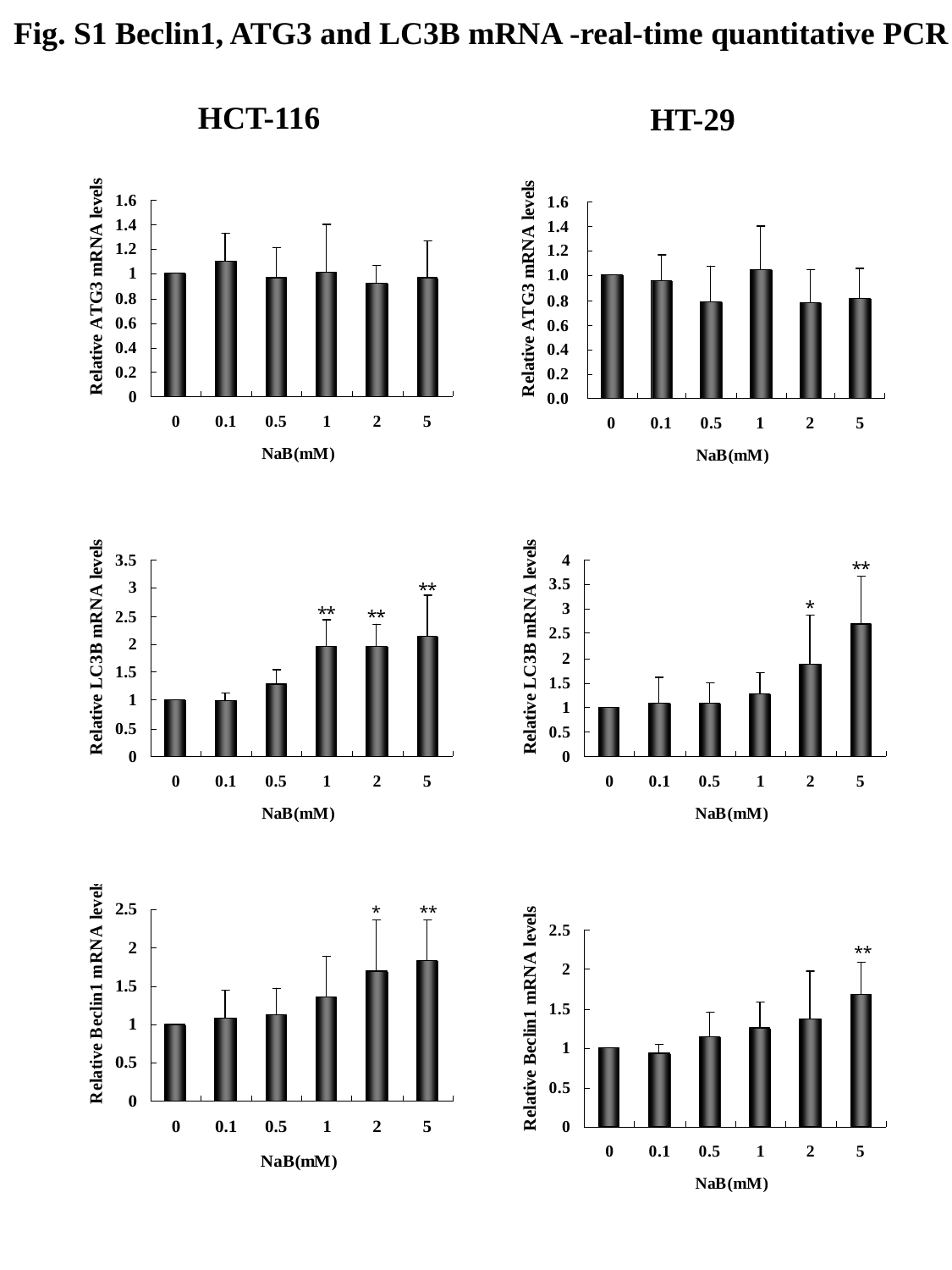

# Fig. S1 Beclin1, ATG3 and LC3B mRNA -real-time quantitative PCR
HCT-116
HT-29

## Slide 2
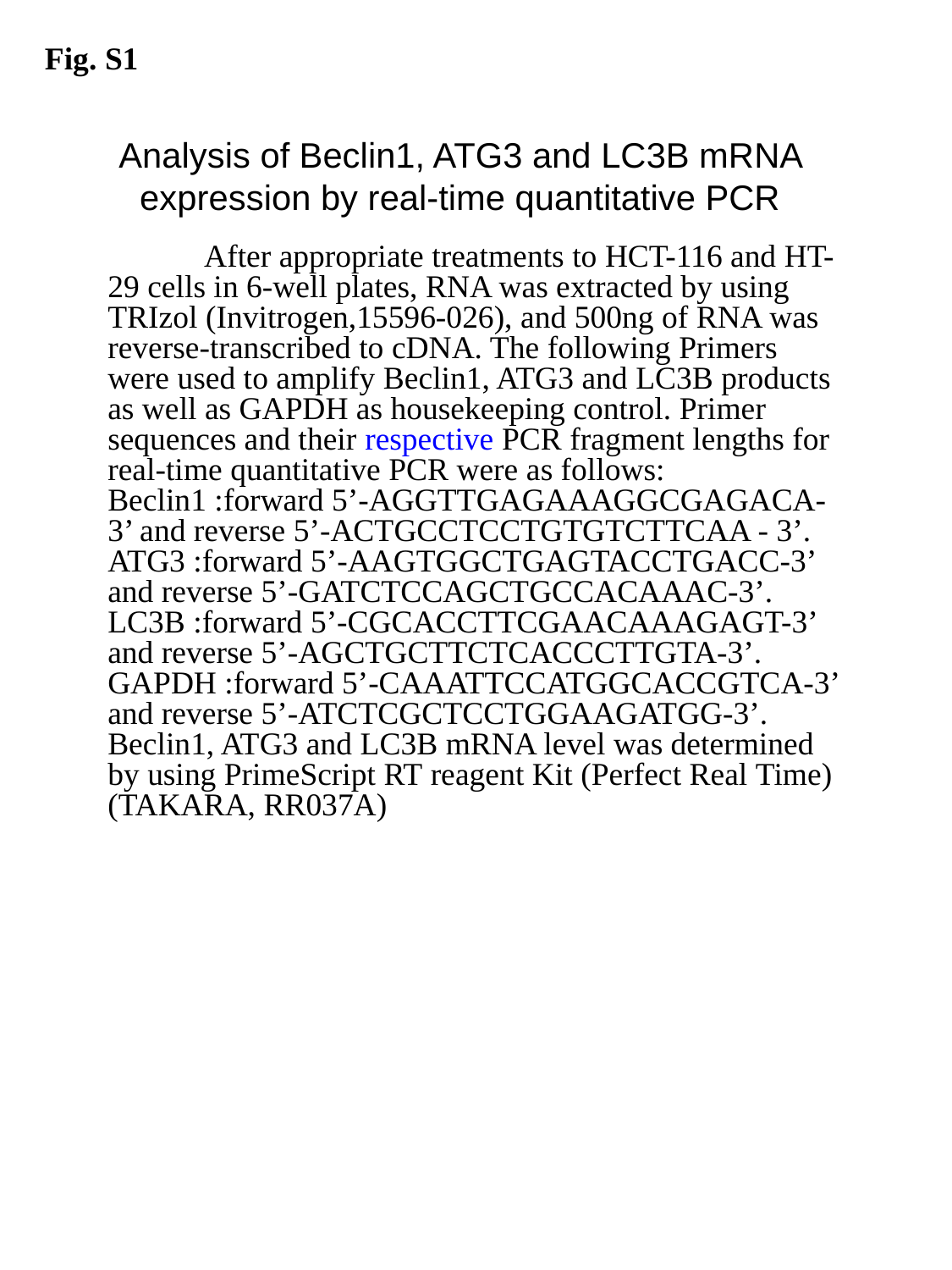

Fig. S1
# Analysis of Beclin1, ATG3 and LC3B mRNA expression by real-time quantitative PCR
 After appropriate treatments to HCT-116 and HT-29 cells in 6-well plates, RNA was extracted by using TRIzol (Invitrogen,15596-026), and 500ng of RNA was reverse-transcribed to cDNA. The following Primers were used to amplify Beclin1, ATG3 and LC3B products as well as GAPDH as housekeeping control. Primer sequences and their respective PCR fragment lengths for real-time quantitative PCR were as follows: Beclin1 :forward 5’-AGGTTGAGAAAGGCGAGACA-3’ and reverse 5’-ACTGCCTCCTGTGTCTTCAA - 3’. ATG3 :forward 5’-AAGTGGCTGAGTACCTGACC-3’ and reverse 5’-GATCTCCAGCTGCCACAAAC-3’. LC3B :forward 5’-CGCACCTTCGAACAAAGAGT-3’ and reverse 5’-AGCTGCTTCTCACCCTTGTA-3’. GAPDH :forward 5’-CAAATTCCATGGCACCGTCA-3’ and reverse 5’-ATCTCGCTCCTGGAAGATGG-3’. Beclin1, ATG3 and LC3B mRNA level was determined by using PrimeScript RT reagent Kit (Perfect Real Time) (TAKARA, RR037A)

## Slide 3
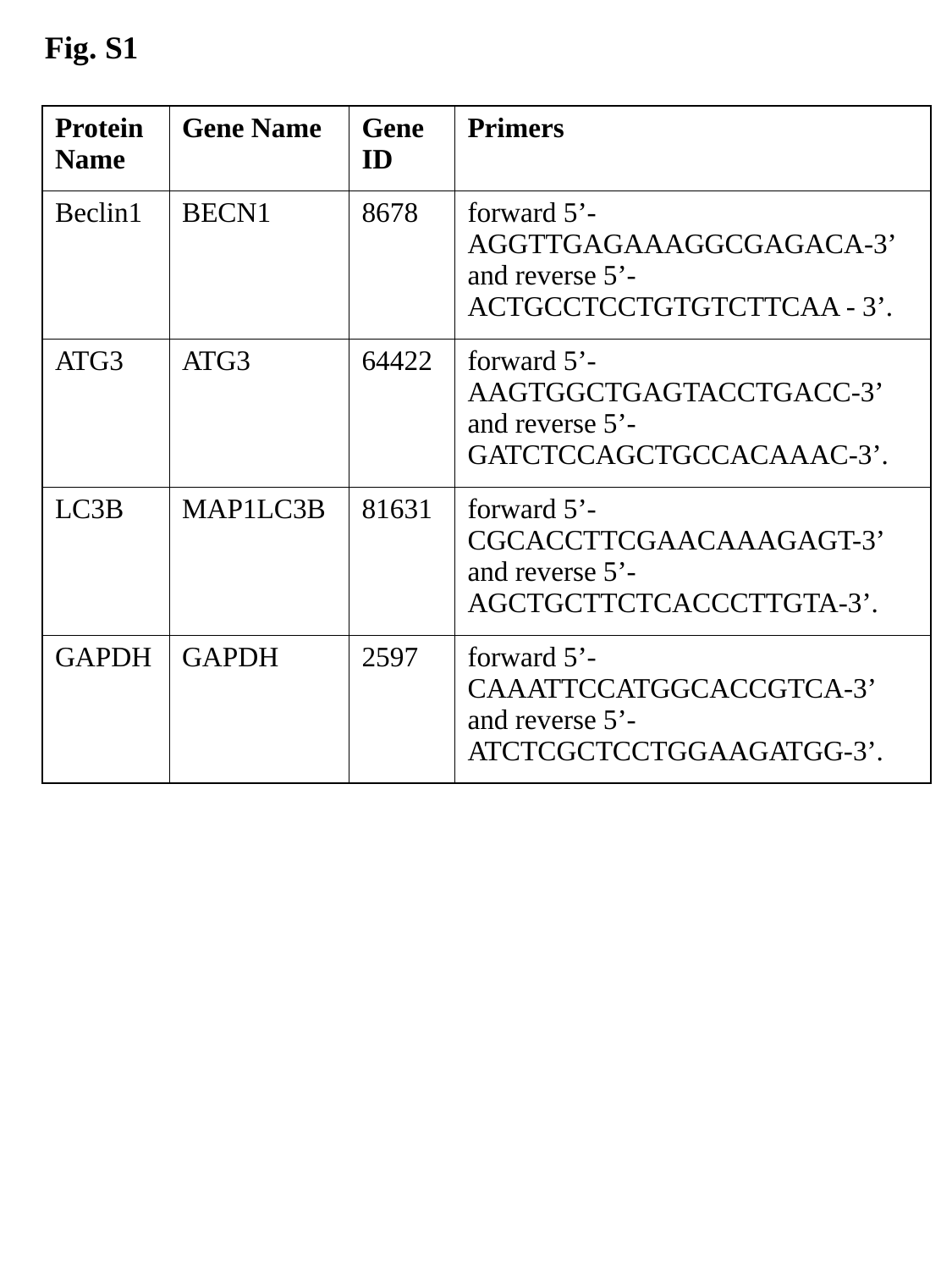

Fig. S1
| Protein Name | Gene Name | Gene ID | Primers |
| --- | --- | --- | --- |
| Beclin1 | BECN1 | 8678 | forward 5’-AGGTTGAGAAAGGCGAGACA-3’ and reverse 5’-ACTGCCTCCTGTGTCTTCAA - 3’. |
| ATG3 | ATG3 | 64422 | forward 5’-AAGTGGCTGAGTACCTGACC-3’ and reverse 5’-GATCTCCAGCTGCCACAAAC-3’. |
| LC3B | MAP1LC3B | 81631 | forward 5’-CGCACCTTCGAACAAAGAGT-3’ and reverse 5’-AGCTGCTTCTCACCCTTGTA-3’. |
| GAPDH | GAPDH | 2597 | forward 5’-CAAATTCCATGGCACCGTCA-3’ and reverse 5’-ATCTCGCTCCTGGAAGATGG-3’. |
#
