# Supplementary material for: Sodium Butyrate Induces Endoplasmic Reticulum Stress and Autophagy in Colorectal Cells: Implications for Apoptosis
Source: PLoS One. 2016 Jan 19;11(1):e0147218. doi: 10.1371/journal.pone.0147218 (PMC4718706; doi:10.1371/journal.pone.0147218)
Supplement: S2 Fig — (A) Representative Western blots showed the PARP expression in HCT-116 or HT-29 cells that were exposed to 0.1μM Mithramycin for 30 minutes followed by treatment with 2mM NaB for 24 hours. GAPDH was used as loading control. (B, C) HCT-116 (B) or HT-29 (C) cells were exposed to 0.1μM Mithramycin for 30 minutes followed by treatment with 2mM NaB for 24 hours in three independent experiments. Flow cytometry showed the percentage of annexin-5/PI (apoptotic cells), which was expressed as the mean ± SD of three independent experiments. One-way ANOVA was used for statistical analysis to compare control cells and NaB treatments. *p<0.05, ** p<0.01 compared to control. (PPT) [file pone.0147218.s002.ppt]

## Slide 1
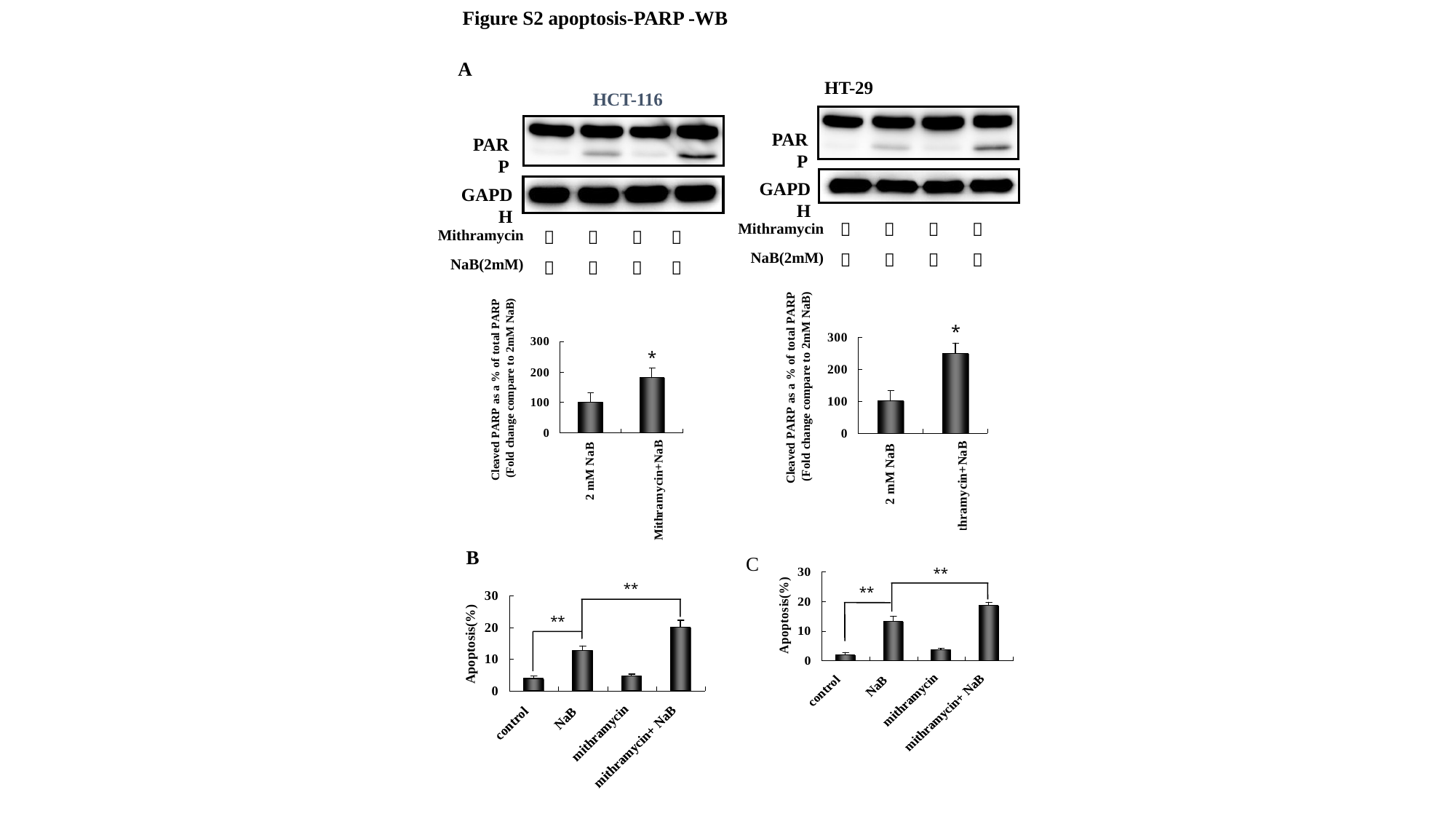

Figure S2 apoptosis-PARP -WB
A
# HT-29
HCT-116
PARP
PARP
GAPDH
GAPDH
Mithramycin
 NaB(2mM)
－ － ＋ ＋
－ ＋ － ＋
Mithramycin
 NaB(2mM)
－ － ＋ ＋
－ ＋ － ＋
**
**
B
**
**
C

## Slide 2
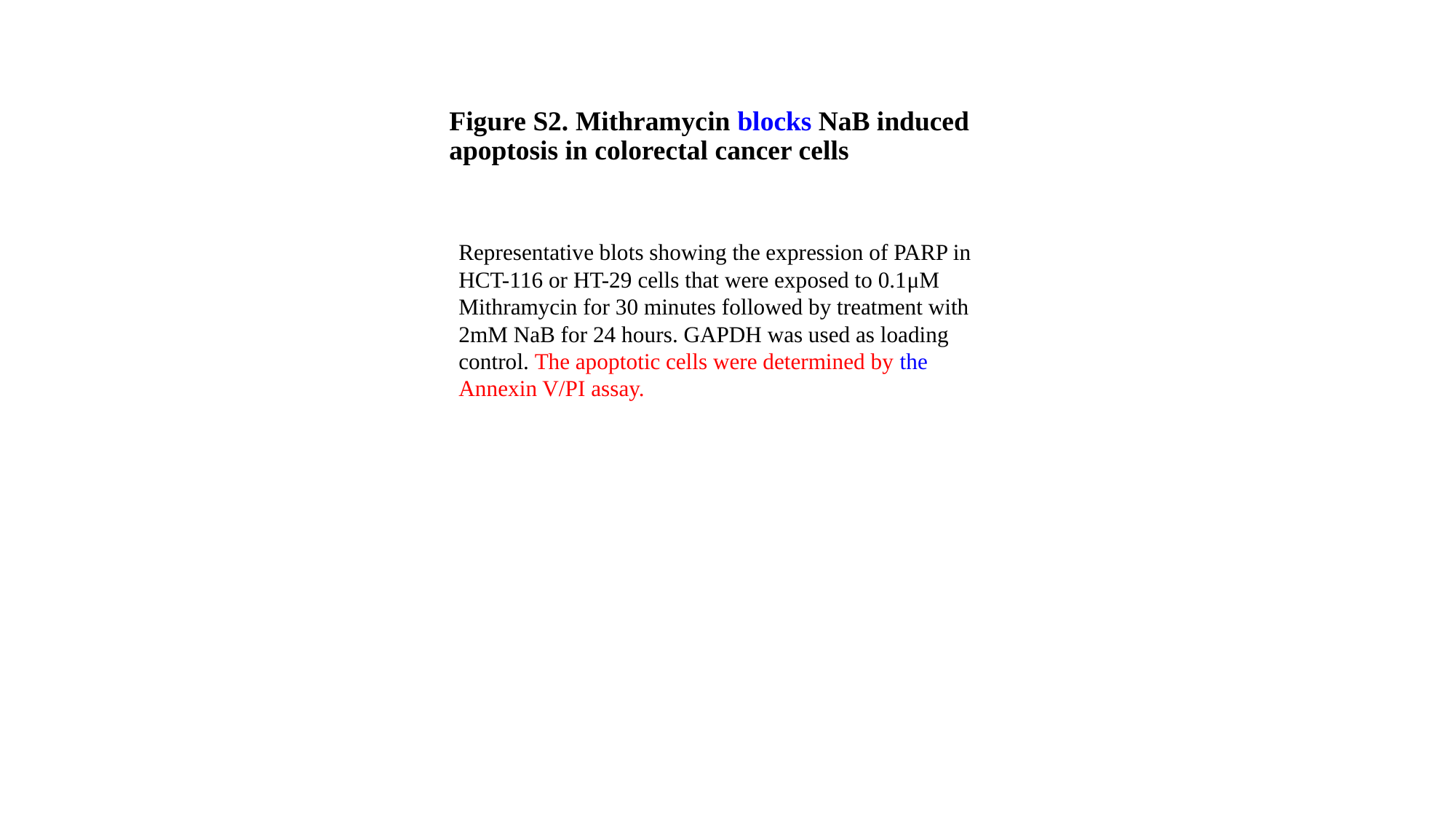

# Figure S2. Mithramycin blocks NaB induced apoptosis in colorectal cancer cells
Representative blots showing the expression of PARP in HCT-116 or HT-29 cells that were exposed to 0.1μM Mithramycin for 30 minutes followed by treatment with 2mM NaB for 24 hours. GAPDH was used as loading control. The apoptotic cells were determined by the Annexin V/PI assay.
