# Supplementary material for: Sodium Butyrate Induces Endoplasmic Reticulum Stress and Autophagy in Colorectal Cells: Implications for Apoptosis
Source: PLoS One. 2016 Jan 19;11(1):e0147218. doi: 10.1371/journal.pone.0147218 (PMC4718706; doi:10.1371/journal.pone.0147218)
Supplement: S1 Table — PCR amplified products were detected using SYBR® Premix Ex Taq™ II (Tli RNaseH Plus) (TAKARA, RR820A). Consistent amplification of DNA was detected by fluorescence of SYBR Green I in real time PCR. (DOC) [file pone.0147218.s003.doc]

S1 Table. Primer sequences for quantitative real-time PCR.

| Protein name | Gene name | Gene ID | Primers | Length of PCR product (bp) |
| --- | --- | --- | --- | --- |
| Beclin1 | BECN1 | 8678 | forward 5’-AGGTTGAGAAAGGCGAGACA-3’ and  reverse 5’-ACTGCCTCCTGTGTCTTCAA - 3’. | 83 |
| ATG3 | ATG3 | 64422 | forward 5’-AAGTGGCTGAGTACCTGACC-3’ and  reverse 5’-GATCTCCAGCTGCCACAAAC-3’ | 93 |
| LC3B | MAP1LC3B | 81631 | forward 5’-CGCACCTTCGAACAAAGAGT-3’ and  reverse 5’-AGCTGCTTCTCACCCTTGTA-3’ | 103 |
| GAPDH | GAPDH | 2597 | forward 5’-CAAATTCCATGGCACCGTCA-3’ and  reverse 5’-ATCTCGCTCCTGGAAGATGG-3’ | 81 |

PCR amplified products were detected using SYBR® Premix Ex Taq™ II (Tli RNaseH Plus) (TAKARA, RR820A). Consistent amplification of DNA was detected by fluorescence of SYBR Green I in real time PCR.
